# Effects of Levodopa-Carbidopa Intestinal Gel on Dyskinesia and Non-Motor Symptoms Including Sleep: Results from a Meta-Analysis with 24-Month Follow-Up

**DOI:** 10.3233/JPD-223295

**Published:** 2022-10-14

**Authors:** K. Ray Chaudhuri, Angelo Antonini, Rajesh Pahwa, Per Odin, Nataliya Titova, Sandeep Thakkar, Sonya J. Snedecor, Saket Hegde, Ali Alobaidi, Juan Carlos Parra, Cindy Zadikoff, Lars Bergmann, David G. Standaert

**Affiliations:** a Parkinson Foundation Centre of Excellence, King’s College Hospital and King’s College, London, UK; b Parkinson and Movement Disorders Unit, Study Center for Neurodegeneration CESNE, Department of Neuroscience, University of Padova, Padova, Italy; c University of Kansas Medical Center, Kansas City, KS, USA; d University of Lund, Lund, Sweden; e N.I. Pirogov Russian National Research Medical University, Moscow, Russia; f Federal State Budgetary Institution «Federal center of brain research and neurotechnologies» of the Federal Medical Biological Agency, Moscow, Russia; g Hoag Hospital Newport Beach, Newport Beach, CA, USA; h OPEN Health, Bethesda, MD, USA; i OPEN Health, New York, NY, USA; j AbbVie Inc., North Chicago, IL, USA; k University of Illinois at Chicago, Chicago, IL, USA; l University of Alabama at Birmingham, Birmingham, AL, USA

**Keywords:** Parkinson’s disease, carbidopa/levodopa, quality of life, motor symptoms, meta-analysis

## Abstract

**Background:**

In advanced Parkinson’s disease (PD), dyskinesias and non-motor symptoms such as sleep dysfunction can significantly impair quality of life, and high-quality management is an unmet need.

**Objective:**

To analyze changes in dyskinesia and non-motor symptoms (including sleep) among studies with levodopa-carbidopa intestinal gel (LCIG) in patients with advanced PD.

**Methods:**

A comprehensive literature review identified relevant studies examining LCIG efficacy. Outcomes of interest were dyskinesia (UDysRS, UPDRS IV item 32), overall non-motor symptoms (NMSS), mentation/behavior/mood (UPDRS I), and sleep/daytime sleepiness (PDSS-2, ESS). The pooled mean (95% confidence interval) change from baseline per outcome was estimated for each 3-month interval with sufficient data (i.e., reported by≥3 studies) up to 24 months using a random-effects model.

**Results:**

Seventeen open-label studies evaluating 1243 patients with advanced PD were included. All outcomes of interest with sufficient data for meta-analysis showed statistically significant improvement within 6 months of starting LCIG. There were statistically significant improvements in dyskinesia duration as measured by UPDRS IV item 32 at 6 months (–1.10 [–1.69, –0.51] h/day) and 12 months (–1.35 [–2.07, –0.62] h/day). There were statistically and clinically significant improvements in non-motor symptoms as measured by NMSS scores at 3 months (–28.71 [–40.26, –17.15] points). Significant reduction of NMSS burden was maintained through 24 months (–17.61 [–21.52, –13.70] points). UPDRS I scores significantly improved at 3 months (–0.39 [–0.55, –0.22] points). Clinically significant improvements in PDSS-2 and ESS scores were observed at 6 and 12 months in individual studies.

**Conclusion:**

Patients with advanced PD receiving LCIG showed significant sustained improvements in the burden of dyskinesia and non-motor symptoms up to 24 months after initiation.

## INTRODUCTION

Parkinson’s disease (PD) is a chronic and progressive disorder primarily associated with the degeneration of brain dopamine neurons and depletion of striatal dopamine [1–3]. The gold standard treatment for PD is levodopa, which is converted to dopamine in the brain [4]. Administration of levodopa orally can lead to motor fluctuations due to its short half-life and delayed or variable gastric emptying [5]. In addition, intermittent or pulsatile dopamine receptor stimulation can lead to postsynaptic plasticity and further drive fluctuations and worsen the motor and non-motor symptoms of PD [2, 5, 6]. Continuous levodopa infusion can be provided in an effort to sustain plasma levels of levodopa, leading to improvement in both motor complications and the shortening response observed with oral levodopa as PD progresses [2, 7–9].

Levodopa-carbidopa intestinal gel (LCIG) is a stable gel suspension suitable for continuous delivery through percutaneous gastrojejunostomy via a portable pump [10]. By continuously delivering medication directly to the jejunum, LCIG can avoid blocks to oral absorption of dopaminergic drugs via delayed gastric emptying and other factors [11], and generate sustained dopamine release and receptor stimulation within a therapeutic window [4–6, 12].

Although motor symptoms traditionally define PD, patients with PD can experience both motor and non-motor symptoms. The development of dyskinesia, a motor symptom, is common in patients with advanced PD [2, 7]. While mild dyskinesia may minimally impact patients, moderate to severe dyskinesia can be disabling and associated with functional impairment, patient discomfort, and social limitations/stigma, as well as reduced quality of life and increased healthcare costs [13–16]. Non-motor symptoms include anxiety, mood disorders, fatigue, cognitive decline and dementia, autonomic dysfunction, and sleep-wake cycle regulation disorders and are an important feature of advanced PD and a chief therapeutic challenge [2, 17]. Approximately 28% of patients with PD rated fluctuations in non-motor symptoms as more disabling than fluctuations in motor symptoms [18]. The burden of non-motor symptoms is a key determinant of quality of life, overall disability progression, and nursing home placement in patients with PD [2, 19, 20]. In particular, sleep and mood are significant predictors of patients reporting poor quality of life [21].

Results from a previously conducted meta-analysis showed improvement in “off” time, activities of daily living (ADLs), and quality of life for patients with advanced PD treated with LCIG persisting up to 24 months [22]. LCIG has been shown to be safe and effective in clinical trials and real-world evidence studies in reducing dyskinesias in patients with advanced PD [6, 23–26], and open-label and global registry studies have shown consistent and robust improvement in several non-motor symptoms, including sleep quality improvement [25, 27–30]. However, the length of follow-up, inclusion criteria, study design, and outcome measures varied among these studies [31]. In addition, there is no previous meta-analysis on non-motor symptoms, sleep, and dyskinesia in advanced PD. Thus, the objective of the present analysis was to pool the available data to assess the impact of LCIG on dyskinesia and non-motor symptoms (such as sleep and mood/behavior) in patients with advanced PD.

## MATERIALS AND METHODS

### Study identification

We conducted a literature review to identify interventional, prospective observational, or retrospective studies published in the Embase and Medline databases through October 6, 2020. Studies of interest were single-arm or comparative evaluations of patients with advanced PD who were initiating LCIG treatment; studies had to report at least 1 outcome of interest and have a follow-up time of at least 3 months. Studies of patients with early PD, case reports, conference abstracts without a full-text publication, and trials or observational studies that did not examine the outcomes of interest were excluded. Following the literature search, 2 reviewers independently screened each title and abstract to identify relevant articles and reviewed the full-text articles for final study inclusion. Disagreements were resolved by discussion and consensus.

### Outcome measures

Outcomes of interest for measuring dyskinesia were the Unified Dyskinesia Rating Scale (UDysRS) and item 32 (modified from percent improvement to hours per day) or items 32–34 (score) of the Unified Parkinson’s Disease Rating Scale (UPDRS) Part IV (UPDRS IV), depending on the study. Outcomes for measuring non-motor symptoms included the Non-Motor Symptoms Scale for Parkinson’s Disease (NMSS), Part I of the UPDRS (UPDRS I) for measuring mood/behavior, the Parkinson’s Disease Sleep Scale-2 (PDSS-2) for sleep, and the Epworth Sleepiness Scale Questionnaire (ESS) for daytime sleepiness.

### Statistical analysis

Data for each outcome were grouped into 3-month intervals (e.g., 1–3 months [“3 months”], 4–6 months [“6 months”], 7–9 months [“9 months”]) up to 24 months. For each outcome of interest, the pooled mean change from baseline (CFB) with 95% CI was estimated for each time interval with sufficient data for meta-analysis (i.e., outcome reported by≥3 available studies). Data were pooled using random-effects methods to generate overall estimates for each outcome and time point (see the Interactive Appendix in the Supplementary Material for fixed-effects results). Heterogeneity among the studies was assessed for each outcome and time point reported by 3 or more studies using the I^2^ and Q statistics.

Outcomes in which the confidence intervals for mean change from baseline do not include zero are statistically significant. Mean changes from baseline that exceed the minimum clinically important differences (NMSS: –13.91 points [32]; PDSS-2: –3.44 points [33]) are considered clinically significant.

Evaluation of the associations between study outcomes and factors such as mean duration of PD, mean age at study entry, Hoehn and Yahr On score, or Hoehn and Yahr Off score showed no impact on differences among the studies; thus, no statistical adjustment for these factors was considered. Further, no formal risk of bias assessment was performed since the conduct of the studies is unlikely to affect the reported outcomes, which are measured with standard instruments and no association between baseline patient characteristics and outcomes was observed. Analyses were conducted using Microsoft Excel software.

## RESULTS

### Study selection and characteristics

**Fig. 1:**
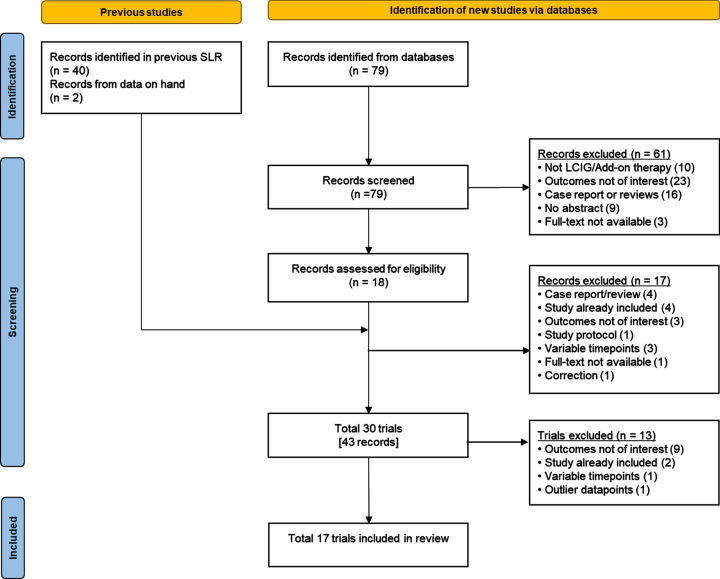
Study selection flow diagram. LCIG, levodopa-carbidopa intestinal gel; SLR, systematic literature review.

A total of 17 studies evaluating a total of 1243 patients with advanced PD were included in the analysis (Fig. 1). No studies were excluded during the similarity assessment. At baseline, mean patient age in the individual studies ranged from 57.8 to 70.6 years and mean PD duration ranged from 10.0 to 16.1 years (Table 1).

**Table 1:** Summary of included studies

Author, Year	Data Source(s)^a^	Study Characteristics			Patient Baseline Characteristics, Mean (SD)				Patient Scores at Baseline, Mean (SD)						
		Study Design	Follow-up, mo^b^	ITT, N	Age, y	PD Duration, y	Daily L-dopa Dose, mg	Hoehn and Yahr Score	UPDRS IV Item 32, h/d	UPDRS IV Items 32–34	UDysRS	NMSS	UPDRS I	PDSS-2	ESS
DUOGLOBE 2020	Publication, CSR [28, 61]	Global, multicountry, single-arm, post-marketing observational analysis	36	164	70.2 (8.17)	11.2 (4.72)	1432 (101.82)	On: 3 (0.83) Off: 3.6 (0.8)	4.1 (3.7)	NA	33.7 (21.14)	88.2 (51.09)	NA	26.6 (11.65)	9.8 (5.26)
Alvarez 2020	Publication, CSR [62, 63]	Open-label, randomized, multicenter interventional study	3	25	69.3 (7.0)	12.7 (4.2)	1211.5 (374.89)	NA	NA	NA	53.2 (12.24)	NA	NA	NA	NA
Antonini 2017	Publication, CSR [27, 64]	Observational non-interventional	24	Retro-spective: 140	67.4 (8.1)	12.6 (6.6)	854.8 (513.3)	On: 2.9 (0.8) Off: 4.1 (0.8)	4.1 (3.7)	NA	NA	76.2 (47.9)	NA	NA	NA
	Prospe-ctive: 189	66.1 (8.5)	13.0 (6.1)	899.7 (474.7)	On: 2.8 (0.8) Off: 3.9 (0.9)	4.4 (3.8)	NA	NA	66.5 (39.6)	NA	NA	NA
Caceres-Redondo 2014	Publication [65]	Observational	24	16	66.5 (9.3)	15.1 (5.4)	1473.0 (449.0)	On: 2.4 (0.5) Off: 3.7 (0.8)	NA	NA	NA	17.3 (4.7)	NA	NA	NA
De Fabregues 2017	Publication [34]	Long-term, open-label, prospective, observational	12	37	68.2 (6.8)	13.5 (5.6)	NA	On: 2.5 Off: 3.8	NA	NA	NA	NA	3.2 (2.4)	NA	5.6 (3.6)
Fasano 2012	Publication [54]	Phase III, open-label	24.9 (14.4)	14	67.1 (11.5)	12.9 (4.8)	929.1 (682.2)	NA	NA	NA	NA	126 (56.18)	8.71 (3.15)	39.08 (8.58)	NA
Fernandez 2015	Publication, CSR, trial registry [66–68]	Phase III, open-label	12	354	64.1 (9.1)	12.5 (5.5)	1082.9 (582.1)	NA	NA	3.7 (2.4)	NA	NA	2.2 (1.9)	NA	NA
Honig 2009	Publication [25]	Prospective open-label observational	6	22	58.6 (9.1)	15.3 (5.9)	NA	Off: 3.8	NA	NA	NA	89.9 (56.5)	NA	NA	NA
Juhasz 2017	Publication [26]	Prospective, multicenter, open-label cohort study	12	34	67.0 (6.0)	12.0 (5.0)	1000.2 (577.6)	3.0	NA	NA	45.9 (16.7)	88.9 (40.3)	NA	27.2 (10.5)	9.1 (4.8)
Kruger 2017	Publication, CSR [69, 70]	Phase III single-arm, open-label, baseline-controlled, multicenter study	12	64	70.4 (7.8)	13.9 (5.4)	NA	NA	NA	NA	NA	95.5 (54.5)	NA	NA	NA
Martinez-Martin 2015	Publication [30]	Prospective, multicenter, open-label cohort study	6	44	62.7 (9.1)	16.1 (6.7)	1815.4 (771.5)	4^c^	NA	NA	NA	90.95 (45)	NA	NA	NA
Olanow 2014	Publication, CSR, 2 trial registries [10, 71–73]	Phase III, double-blind RCT	3	37	63.7 (9.5)	10.0 (4.6)	1005.4 (373.6)	On: 2.3 (0.6)	NA	2.4 (1.6)	NA	NA	1.8 (1.7)	NA	NA
Palhagen 2016	Publication, CSR, trial registry [74–76]	Prospective, open-label, long-term study	36	27	64.6 (6.4)	10.7	NA	On: 2.0 (0.9) Off: 3.6 (1.1)	NA	NA	NA	NA	2.9 (1.9)	NA	NA
Reddy 2012	Publication [77]	Single-center, real-life setting, matched based on PCT funding restrictions	6	17	57.82 (7.71)	16.12 (5.84)	1996 (675)	On: 3.5	NA	NA	NA	113.88 (49.26)	NA	NA	NA
Sensi 2014	Publication [78]	Prospective open-label cohort study	24	17	67.6 (6.1)	15.5 (4.0)	1158.9 (334.5)	On: 3.2 (0.7)	NA	NA	NA	51.8 (37.3)	NA	NA	NA
Slevin 2015	Publication, trial registry [79, 80]	Phase III, open-label, multicenter	12	29	64.8 (6.6)	11.4 (5.7)	NA	NA	NA	NA	NA	NA	1.2 (1.0)	NA	NA
Standaert 2017	Publication, CSR, trial registry [6, 81, 82]	Phase IIIb, open-label	12	38	64.3 (10.2)	11.5 (5.3)	NA	NA	NA	NA	NA	48.3 (35.6)	1.6 (1.6)	NA	NA

CSR, clinical study report; ESS, Epworth Sleepiness Scale; ITT, intent to treat; L-dopa, levodopa; N, number; NA, not available; NMSS, Non-Motor Symptoms Scale for Parkinson’s Disease; PCT, primary care trust; PD, Parkinson’s disease; PDSS-2, Parkinson’s Disease Sleep Scale-2; RCT, randomized controlled trial; UDysRS, Unified Dyskinesia Rating Scale; UPDRS, Unified Parkinson’s Disease Rating Scale. ^a^The individual record(s) used per study are cited in this column. ^b^Values in this table express the follow-up period defined in study publication(s). If a study did not report a prespecified follow-up period, but did report mean (SD) follow-up time, the mean (SD) follow-up time reported is presented in this table. ^c^Median score, not mean.

### Outcomes

All outcomes of interest with sufficient data for meta-analysis showed statistically significant improvement within 6 months of starting LCIG treatment (Table 2). Meta-analysis was possible for UPDRS IV modified item 32 (hours per day) outcome scores at 4 time points, NMSS scores at 5 time points, and UPDRS I scores at 2 time points. Even for time points where meta-analysis was not possible, the majority of results from individual studies showed improvement in the evaluated outcomes.

The results of our meta-analysis showed statistically significant improvements in dyskinesia duration as measured by UPDRS IV item 32 starting at 6 months (mean [95% CI] CFB, –1.10 [–1.69, –0.51] h/day) that was sustained through 24 months (–0.76 [–1.31, –0.22] h/day). Changes from baseline in UDysRS and UPDRS IV items 32–34 scores were not meta-analyzed, as there were no 3-month intervals in which 3 or more studies reported these outcomes. However, among the individual studies, improvements in UDysRS and UPDRS IV items 32–34 scores were observed at 3 months, 6 months, and 12 months.

**Fig. 2:**
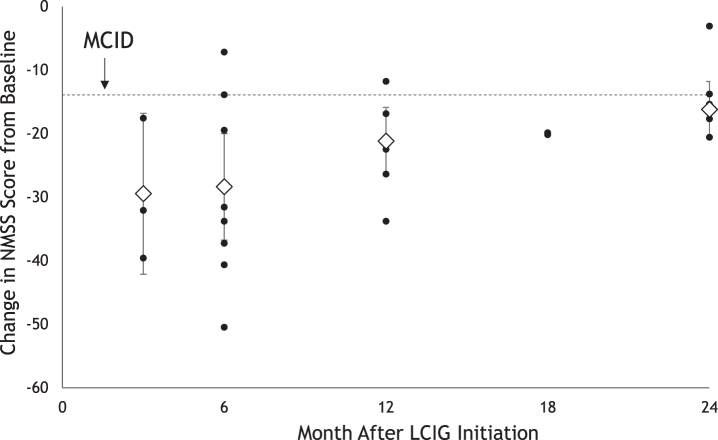
Impact of LCIG on Non-Motor Symptoms Up to 24 Months After Initiation^a^. LCIG, levodopa-carbidopa intestinal gel; MCID, minimum clinically important difference; NMSS, Non-Motor Symptoms Scale. ^a^Black circles represent reported mean change from baseline. Diamonds represent pooled mean change from baseline with 95% CIs estimated using random effects meta-analysis.

Non-motor symptoms as measured on the NMSS scale showed statistically and clinically significant improvement starting at 3 months (mean [95% CI] CFB, –28.71 [–40.26, –17.15] points) that was sustained through 24 months (–17.61 [–21.52, –13.70] points) (Fig. 2).

**Table 2:** Impact of LCIG on dyskinesia, non-motor symptoms, and sleep outcomes in patients with advanced PD

Symptom Domain	Outcome Measure	MCID	Score Range	Change From Baseline at Time Interval^a^				
				1 to 3 Months	4 to 6 Months	10 to 12 Months	16 to 18 Months	22 to 24 Months
Dyskinesia	UPDRS IV, item 32 (h/day)	NA	0 (best) to 24 (worst)	*n* = 99	*n* = 422	*n* = 363	*n* = 140, *n* = 189	*n* = 140, *n* = 189
				–0.7 (4.77)^[28] ,c^	**–1.10 (–1.69, –0.51)**	**–1.35 (–2.07, –0.62)**	**–0.77 (–1.31, –0.22)**	**–0.76 (–1.31, –0.22)**
	UPDRS IV, items 32–34 (score)	NA	0 (best) to 12 (worst)	*n* = 34, *n* = 275	*n* = 267	*n* = 250
				–0.2 (1.9)^[10] ,c^ –1.4 (0.2)^[68] ,b^	–1.2 (0.2)^[68] ,b^	–1.2 (0.2)^[68] ,b^	No data	No data
	UDysRS score	NA	0 (best) to 104 (worst)	*n* = 24, *n* = 91	*n* = 83	*n* = 34, *n* = 26
				–17.4 (2.79)^[63] ,b^ –11.9 (18.92)^[28] ,c^	–12.5 (19.91)^[28] ,c^	–13.8 (NR)^[26]^ –9.9 (22.73)^[28] ,c^	–9.00 (21.69)^[28] ,c^	–8.1 (22.57)^[28] ,c^
Non-motor symptoms	NMSS score	–13.91^[32]^	0 (best) to 243 (worst)	*n* = 174	*n* = 561	*n* = 459	*n* = 178, *n* = 189	*n* = 376
				**–28.71 (–40.26, –17.15)**	**–27.81 (–35.73, –19.89)**	**–20.97 (–25.85, –16.09)**	**–18.84 (–22.56, –15.12)**	**–17.61 (–21.52, –13.70)**
Mood/ Behavior	UPDRS I score	NA	0 (best) to 16 (worst)	*n* = 407	*n* = 267, *n* = 27	*n* = 339	*n* = 27	*n* = 14, *n* = 27
				**–0.39 (–0.55, –0.22)**	–0.2 (0.1)^[68] ,b^ –0.1 (1.7)^[76] ,c^	**0.11 (–0.20, 0.42)**	1.1 (3.1)^[76] ,c^	–1.92 (NR)^[54]^ 1.0 (3.0)^[76] ,c^
Sleep	PDSS-2 score	–3.44^[33]^	0 (best) to 60 (worst)	*n* = 106	*n* = 99	*n* = 34, *n* = 37	*n* = 14
				–7.2 (13.35)^[28] ,c^	–8.1 (13.09)^[28] ,c^	–4.0 (NR)^[26]^ –6.6 (12.19)^[28] ,c^	–5.9 (12.34)^[28] ,c^	–5.62 (NR)^[54]^ –5.80 (13.15)^[28] ,c^
	ESS score	NA	0 (best) to 24 (worst)	*n* = 107	*n* = 37, *n* = 100	*n* = 34, *n* = 36
				–1.5 (5.12)^[28] ,c^	–2.8 (NR)^[34]^ –1.2 (5.66)^[28] ,c^	–1.0 (NR)^[26]^ –1.0 (5.73)^[28] ,c^	–1.10 (6.08)^[28] ,c^	–1.40 (6.11)^[28] ,c^

ESS, Epworth Sleepiness Scale; LCIG, levodopa-carbidopa intestinal gel; MCID, minimum clinically important difference; NA, not available; NMSS, Non-Motor Symptoms Scale; NR, not reported; PD, Parkinson’s disease; PDSS-2, Parkinson’s Disease Sleep Scale-2; UDysRS, Unified Dyskinesia Rating Scale; UPDRS, Unified Parkinson’s Disease Rating Scale. ^a^**Bold** values in these columns indicate meta-analysis outcomes, which are reported as mean (95% CI) change from baseline and were estimated using random effects analysis. When fewer than 3 studies were available, meta-analysis was not performed. Insufficient data for meta-analysis were available for any outcome for the 7–9 month, 13–15 month, and 19–21 month time intervals. ^b^Results shown in table are mean (SE) values as reported in individual source publications. See citations in cells for specific source publication(s) referenced. Further information on values from individual source studies is presented in the Interactive Appendix. ^c^Results shown are mean (SD) values as reported in individual source publications.

Mentation, behavior, and mood as measured by UPDRS I scores was significantly improved at 3 months (mean [95% CI] CFB, –0.39 [–0.55, –0.22] points). No statistically significant CFB in UPDRS I scores was observed at 12 months (0.11 [–0.20, 0.42] points). There were insufficient data for meta-analysis for the 4 to 6 month, 16 to 18 month, and 22 to 24 month intervals, but among the individual studies, improvements were observed at 6 months and 24 months, with no improvement observed at 18 months.

Sleep outcomes as measured by PDSS-2 and ESS scores were not meta-analyzed, as there were no 3-month intervals in which 3 or more studies reported these outcomes. However, among the individual studies, clinically significant improvements in PDSS-2 scores were observed at 3 months [28], 6 months [28], 12 months [26, 28], 18 months [28], and 24 months [28]. For ESS scores, improvements were observed at 3 months [28], 6 months [28, 34], 12 months [26, 28], 18 months [28], and 24 months [28], with clinically significant improvement at 6 months and 12 months.

Forest plots showing input data from individual studies along with the meta-analysis outcomes are presented in the Interactive Appendix.

## DISCUSSION

Our analysis including data from 17 clinical and observational studies provides one of the most comprehensive assessments to date of the impact of LCIG therapy on non-motor symptom and dyskinesia outcomes among patients with advanced PD. Results of the meta-analysis suggest that the effects of LCIG on non-motor symptoms and dyskinesia are evident as early as 1 to 3 months and 4 to 6 months following treatment initiation, respectively, and are sustained over a period of 24 months. The improvement in non-motor symptoms from baseline was statistically significant and greater than the reported minimum clinically important difference for NMSS scores (–13.91) across 24 months [32]. Mood and behavior was significantly improved at 3 months. In most outcomes, the available data showed a pattern of initial improvement followed by a gradual worsening. This pattern was also observed in quality of life and ADL outcomes [22], which could be attributed to disease progression. However, most measures still remain improved or significantly improved over baseline at 24 months.

Although multiple treatments address dyskinesia in advanced PD, there is an unmet need for therapies to address the substantial burden of non-motor symptoms [2, 19, 20]. The results of our meta-analysis provide an important summary of the published evidence on the effect of LCIG on non-motor symptoms in advanced PD. Our analysis included 12 studies reporting NMSS scores in patients with PD. The statistically significant and clinically meaningful improvement in NMSS scores observed with LCIG in our analysis is consistent with the robust non-motor symptom effects observed in a systematic literature review of multiple large multicenter and observational studies, specifically in the sleep, mood, and fatigue domains [31]. Some recent studies such as INSIGHTS are not included in our analysis due to the study identification cut-off date. However, the changes from baseline to 6 months reported in this trial for NMSS and PDSS-2 were consistent with our estimates [35].

The results of our meta-analysis suggest the use of LCIG for sleep and other key non-motor symptoms of PD. While pathophysiology for improvements in non-motor symptoms is not fully understood, several domains may be partly driven by dopamine control [36]. These domains include depression, sleep dysfunction such as insomnia, restless legs syndrome, nocturia, pain, sexual dysfunction, apathy, and anhedonia [25, 36, 37]. The link between sleep dysfunction and dopamine pathophysiology can also be supported by reduced binding of 11 C-raclopride in the hypothalamus among PD patients, which is a key sleep and autonomic regulatory center [38]. Switching to LCIG would allow reduction in other anti-parkinsonian medications and potentially achieving monotherapy during the day [39]. As result, reduction in other dopaminergic medications may lead to improvements in other non-motor symptoms such as insomnia, sedation and urinary urgency [25]. Improvements in motor symptoms at night (as measured by PDSS-2) may also be correlated with a reduction in daytime sleepiness (as measured by ESS). Lastly, LCIG treatment can be maintained during the night when medically justified. This can lead to improvements in dopamine-driven non-motor symptoms including sleep [25, 40]. Therefore, improvement in dopamine-driven non-motor symptoms is likely to be associated with continuous and stable delivery of levodopa with LCIG compared to orals.

In observational studies, improvement in non-motor symptoms and dyskinesias was associated with improved quality of life in patients with advanced PD receiving LCIG treatment as well as their caregivers [19, 41]. In a cross-sectional epidemiologic study conducted in Italy, caregiver burden also tended to be lower when patients were treated with LCIG compared with continuous subcutaneous apomorphine infusion or continuation of standard of care [42]. Our previous meta-analysis showed that improvement in ADLs and quality of life for patients with advanced PD receiving LCIG persisted up to 24 months [22].

Results from this meta-analysis show statistically and clinically significant improvements in non-motor symptoms and a statistically significant reduction in dyskinesia duration, which contribute to LCIG’s overall impact in improving patients’ quality of life and ability to perform ADLs. Few studies assessed dyskinesia disability (UPDRS IV item 33, UDysRS), leading to limited evidence and an inability to evaluate these outcomes in meta-analysis. PD is associated with substantial economic burden to patients, caregivers, payers, and society [43–46]; improvements in patient quality of life could in turn reduce caregiver burden and improve caregiver quality of life [47]. Informal care costs are a large component of total costs [44, 46, 48], so potential reductions in caregiver burden and use of informal care could impact the total economic toll of PD.

However, while LCIG has been shown to improve dyskinesia, it is worth noting that breakthrough dyskinesia has been described in the long term, although this occurs only in a minority of patients [49]. Few patients may experience exacerbation of levodopa-induced dyskinesia after LCIG initiation [49–53], which can present in different forms including “peak of dose,” “on” period, or diphasic dyskinesia. Monitoring of patient symptoms and tailoring LCIG dose are warranted to insure optimal outcomes during LCIG initiation.

Although there were insufficient data to meta-analyze sleep outcomes in our analysis, clinically significant improvements with LCIG were observed in at least 1 study at 3, 6, 12, 18, and 24 months for PDSS-2 scores [26, 28, 54], and at 6 months and 12 months for ESS scores [26, 28, 34]. Improvements in sleep outcomes could also affect patients’ and caregivers’ daily lives, as patient sleep quality is significantly correlated with caregiver burden, quality of life, and sleep quality [47].

To our knowledge, there has been only one other meta-analysis to date evaluating LCIG in patients with advanced PD that included any of the outcomes used in our analysis. Liu et al. found significant improvement for subthalamic nucleus deep-brain stimulation versus LCIG for dyskinesia/motor fluctuation, measured using overall UPDRS IV scores [55]. These results are consistent with those of our meta-analysis, which evaluated UPDRS IV item 32 and items 32–34 and UDysRS total score change. However, our study focused on the efficacy of LCIG in improving outcomes versus baseline. The comparative efficacy of LCIG versus other treatment options for advanced PD was not assessed in this study.

Our meta-analysis presents a robust evaluation of the impact of LCIG on key PD-related outcomes over 24 months, overcoming the challenge of sample size in individual studies. However, as in all meta-analyses, our evaluation pools data from multiple studies with different study characteristics. We have closely examined the patient populations and outcome definitions to evaluate the impact these factors may have on LCIG treatment outcomes and to create a collection of studies with sufficient similarity to be suitable for meta-analytic combination. No studies were excluded during the similarity assessment.

A tailored, multidisciplinary approach to treatment decisions is recommended in advanced PD [4, 56–58], and the modern approach concentrates on personalized precise delivery of care using the circle of personalized medicine [59]. The choice of personalized therapy in PD is driven by motor and non-motor symptoms, comorbidities, age, caregiver support, and patient/caregiver preferences [60]. Improvements in patients’ daily functions and quality of life are a key component of treatment decision making [56], so the significant improvement in dyskinesia and non-motor symptoms with LCIG in this meta-analysis and the potential impact on patient and caregiver quality of life could influence healthcare providers’ treatment choices.

### Conclusions

In this large-scale meta-analysis involving 1243 unique patients with advanced PD treated with LCIG, we found significant improvement in dyskinesia duration from 6 through 24 months and in non-motor symptoms from 3 through 24 months after treatment initiation. Improvement in NMSS scores was both statistically significant and clinically meaningful. Improvement in UPDRS IV and NMSS was rapid, appearing within the first 6 months, and remained consistent for up to 24 months. LCIG provided improvement in dyskinesia and NMSS including sleep and mood scores. The data available on other sleep endpoints were insufficient for meta-analysis, and additional studies are needed to evaluate the impacts of LCIG on sleep parameters in patients with PD.

## Supplementary Material

Supplementary Material
