# Supplementary material for: Effects of Levodopa-Carbidopa Intestinal Gel on Dyskinesia and Non-Motor Symptoms Including Sleep: Results from a Meta-Analysis with 24-Month Follow-Up
Source: J Parkinsons Dis. 2022 Oct 14;12(7):2071–83. doi: 10.3233/JPD-223295 (PMC9661331; doi:10.3233/JPD-223295)
Supplement: Supplementary Material [file jpd-12-jpd223295-s001.html]

Duodopa Manuscript Web Appendix
**We're sorry but test doesn't work properly without JavaScript enabled. Please enable it to
continue.**
